# Phage Therapy for Mosquito Larval Control: a Proof-of-Principle Study

**DOI:** 10.1128/mbio.03017-22

**Published:** 2022-11-29

**Authors:** Chinmay V. Tikhe, George Dimopoulos

**Affiliations:** a W. Harry Feinstone Department of Molecular Microbiology and Immunology, Johns Hopkins Bloomberg School of Public Health, Baltimore, Maryland, USA; b Johns Hopkins Malaria Research Institute, Baltimore, Maryland, USA; University of Hawaii at Manoa

**Keywords:** *Anopheles*, bacteriophage, bacteriophage therapy, larval development

## Abstract

The mosquito microbiota has a profound impact on multiple biological processes ranging from reproduction to disease transmission. Interestingly, the adult mosquito microbiota is largely derived from the larval microbiota, which in turn is dependent on the microbiota of their water habitat. The larval microbiota not only plays a crucial role in larval development but also has a significant impact on the adult stage of the mosquito. By precisely engineering the larval microbiota, it is feasible to alter larval development and other life history traits of the mosquitoes. Bacteriophages, given their host specificity, can serve as a tool for modulating the microbiota. For this proof-of-principle study, we selected representative strains of five common *Anopheles* mosquito-associated bacterial genera, namely, Enterobacter, Serratia, Pseudomonas, Elizabethkingia, and Asaia. Our results with monoaxenic cultures showed that *Anopheles* larvae with Enterobacter and Pseudomonas displayed normal larval development with no significant mortality. However, monoaxenic *Anopheles* larvae with *Elizabethkingia* showed delayed larval development and higher mortality. *Serratia* and *Asaia* gnotobiotic larvae failed to develop past the first instar. We isolated and characterized three novel bacteriophages (EP1, SP1, and EKP1) targeting Enterobacter, *Serratia,* and *Elizabethkingia,* respectively, and utilized a previously characterized bacteriophage (GH1) targeting Pseudomonas to modulate larval water microbiota. Gnotobiotic *Anopheles* larvae with all five bacterial genera showed reduced survival and larval development with the addition of bacteriophages EP1 and GH1, targeting Enterobacter and Pseudomonas, respectively. The effect was synergistic when both EP1 and GH1 were added together. Our results demonstrate a novel application of bacteriophages for mosquito control.

## INTRODUCTION

Microbes play key roles in the biology of mosquitoes. In the adult stages of the mosquito, gut bacteria are important for blood digestion and egg production (1, 2). Mosquito-associated bacteria have been shown to alter mosquito vector competence (3 to 5). In addition, bacteria in the midgut also affect the mating preferences of mosquitoes (6). Interestingly, the microbiota in the adult mosquitoes is preliminarily derived from their larval stages and water habitat.

The mosquito life cycle begins when a female mosquito lays eggs in or around a body of water. The eggs hatch into larvae and encounter microbes from the breeding water. The microbiota has been shown to be essential for larval development under natural conditions (1, 7 to 9). It was recently demonstrated that microbes present in the breeding water provide the larvae with the essential nutrient riboflavin (10, 11). This dependency on microbes for larval development has been observed in diverse mosquito species (9). It is also important to note that not all bacteria found in larval breeding water can support normal larval development (1). The type and density of bacteria in the water also have a major impact on the larval development rate (8, 12, 13). In addition to larval development, the mosquitoes’ larval microbiota has a major impact on their adult life-history traits.

Bacteria in the breeding water affect the size of adult mosquitoes and their egg production (1, 14 to 16). Bacterial exposure in the larval stage has been shown to alter immune gene expression and the response to arboviral infection in the adult stage (16, 17). Overall, larvae–microbiota interactions can have a profound impact on the adult stages of mosquitoes. Given the immense impact of interactions with the larval microbiota on mosquito biology, these interactions can potentially be exploited to alter the mosquitoes’ life-history traits.

Interestingly, the larval microbiota is largely derived from the surrounding water. Multiple studies have shown that mosquito larvae from different collection sites have diverse microbiota (9, 18 to 20). These studies indicate that larvae–microbiota interactions are largely driven by the environment, and mosquitoes are not facultatively dependent on specific bacteria (9, 21). However, not all the bacteria found in the breeding water can colonize the larvae. Mosquito larvae are hypothesized to act as bacterial filters, with only certain bacteria being able to colonize the larval gut and become part of the adult microbiota (1, 19). Different mosquito species grown in the same breeding water also show differences in their microbial composition, suggesting that microbial colonization is species-specific (22). Despite these differences, certain species of bacteria have been observed to be consistently associated with mosquito species, suggesting the existence of some sort of commensal or symbiotic association (23 to 26). Although multiple studies have referred to mosquito-associated bacteria as symbionts, this role has not yet been fully established.

Despite multiple studies, we know of no instance in which the larval microbiota has been utilized as a target to modulate the life-history traits of mosquitoes. Most of the mosquito larvae–microbiota studies to date have involved inoculating a single strain of bacteria into the water. However, to study the complex interactions between the bacterial members of the microbiota, a targeted modulation of the microbiota is desirable.

Bacteriophages can serve as an ideal tool for targeted modulation of microbial communities (27). They offer several advantages. (i) They offer target specificity: given their host specificity, bacteriophages can be used to selectively reduce the amount of a desired bacterial species within a complex microbiota. (ii) Because of their self-replication, application of one dose is sufficient. (iii) Bacteriophages are environmentally friendly, and many bacteriophage-based products have already been approved for the food industry (28, 29). Bacteriophages are widely used for phage therapy to treat bacterial infections, especially antibiotic-resistant infections (30); they have also been used successfully in a mouse model system to modulate the gut microbiota of the mice (31). In this case, bacteriophage-induced dysbiosis resulted in a cascade of changes in microbial composition and metabolomics in the mice (31). These studies highlight the utility of bacteriophage-based targeted modulation of the host-associated microbiota and its effect on the host.

In this proof-of-principle study, we selected representative members of five bacterial genera, namely, Enterobacter, Pseudomonas, *Serratia*, *Elizabethkingia*, and *Asaia*, that are commonly associated with the primary African malaria vector *An. gambiae* (26). We isolated and characterized three novel bacteriophages targeting Enterobacter, *Serratia*, and *Elizabethkingia*. Along with these new bacteriophages, we utilized a previously characterized Pseudomonas bacteriophage for targeted microbiota modulation. Larval development and survival assays of the individual bacterial taxa showed that of the five genera, only Enterobacter and Pseudomonas supported normal larval development and survival. We then performed larval development and survival assays with an artificial bacterial community comprising all five bacterial genera. When bacteriophages targeting Enterobacter and Pseudomonas were added to the larval water, we observed increased larval mortality and reduced development success. Our results demonstrate the utility of bacteriophages for mosquito microbiota research and raise the possibility of developing phage therapy for mosquito larval control.

## RESULTS

Of the five bacteria, Enterobacter, Pseudomonas, *Serratia*, *Elizabethkingia*, and *Asaia*, we were able to isolate bacteriophages infecting all the bacteria except *Asaia*. All the Pseudomonas bacteriophages isolated were highly unstable under the storage conditions tested here (4°C, −20°C, and −80°C), and so a continuously infecting stock of Pseudomonas bacteriophages could not be maintained. For this reason, for our experiments we utilized a previously well-characterized, highly lytic bacteriophage, GH1, and its host, which is an environmental isolate of Pseudomonas putida (ATCC 12633). For *Asaia*, we tested *Gluconobacter* phage Werquin; however, it was unable to infect *Asaia*. The newly isolated Enterobacter bacteriophage was designated EP1, the *Serratia* bacteriophage was designated SP1, and the *Elizabethkingia* bacteriophage was designated EKP1.

### Characterization of Enterobacter phage EP1.

Enterobacter phage EP1 produced large clear plaques of ~2 to 3 mm on its host Enterobacter sp., suggesting a lytic life cycle. Transmission electron microscopy of the phage particles showed that phage EP1consisted of an icosahedral capsid, ~50 to 55 nm in diameter, and a noncontractile tail (Fig. S1A). Because of this typical morphology, phage EP1 was classified as a member of the *Podovirida*e family. VIRFAM analysis also classified phage EP1 as a Podoviridae of Type 3.

10.1128/mbio.03017-22.1 FIG S1 Transmission electron micrographs of newly isolated bacteriophages: (A) Enterobacter phage EP 1; (B) *Serratia* phage SP1; and C) *Elizabethkingia* EKP1. Download FIG S1, TIF file, 0.9 MB. Copyright © 2022 Tikhe and Dimopoulos. 2022 Tikhe and Dimopoulos. https://creativecommons.org/licenses/by/4.0/ This content is distributed under the terms of the Creative Commons Attribution 4.0 International license.

**FIG 1:**
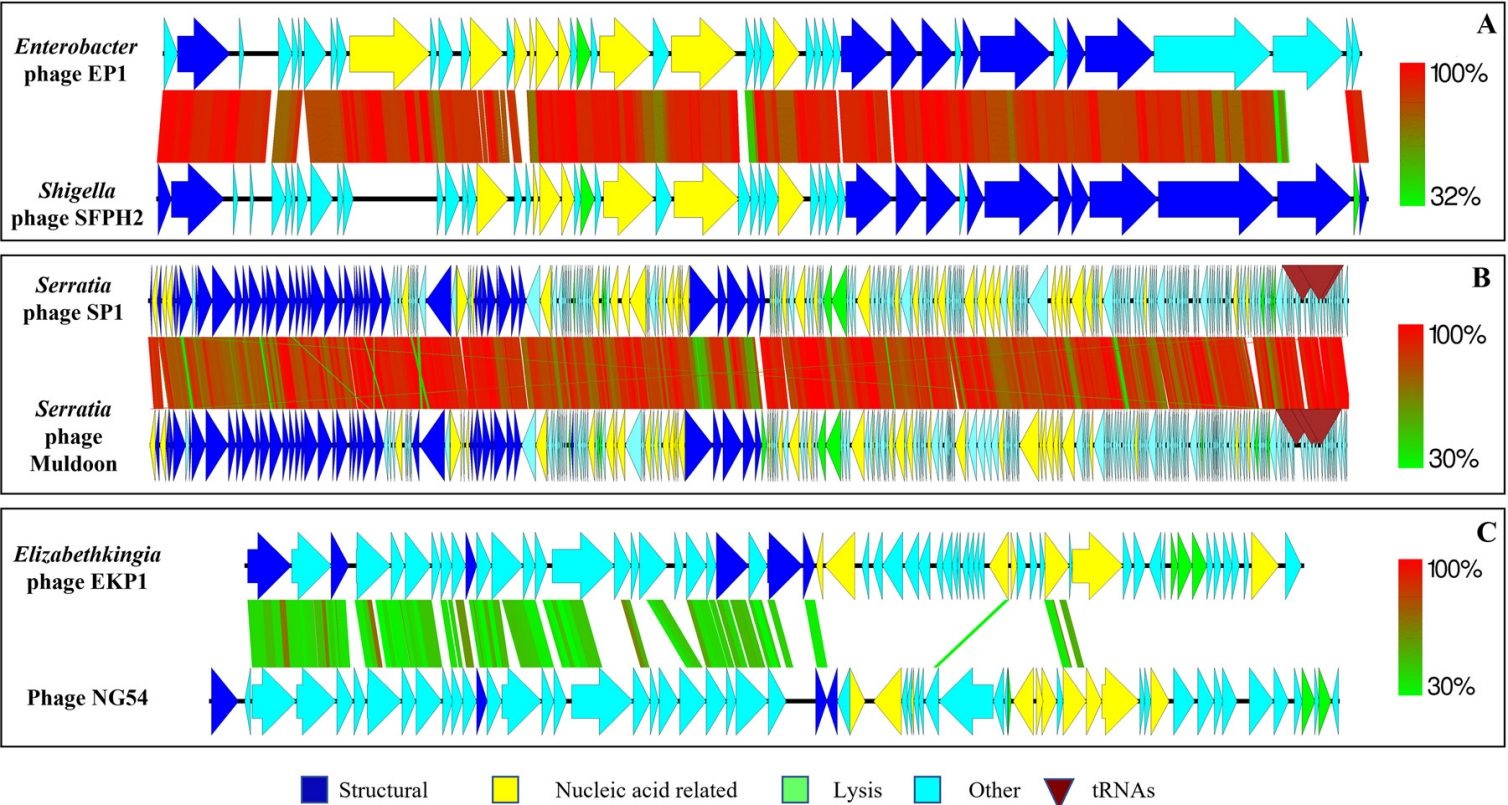
Comparative genomics of newly isolated and characterized bacteriophages with their closest matching bacteriophages from the NCBI GenBank database. (A) Enterobacter phage EP1; (B) *Serratia* phage SP1; (C) *Elizabethkingia* phage EKP1. The heat scale depicts the amino acid similarity (percentage) of predicted protein coding sequences with the syntenous bacteriophage genome. The figure was generated with Easyfig.

Genome sequencing of Enterobacter phage EP1 revealed a 39,940-bp linear double-stranded DNA genome with 52.10% GC content. The EP1 genome was predicted to have 45 putative protein coding open reading frames (ORFs). Of the 45 ORFs, 21 (46.66%) were predicted to be hypothetical proteins with no known assigned function. Twelve ORFs were predicted to be phage structural proteins, nine were predicted to have DNA-related functions, and three were assigned bacterial cell lysis-related functions. Phage EP1 did not have any tRNAs in its genome (Fig. 1A).

Phage EP1 genome showed high similarity at the nucleotide level to *Shigella* phage SFPH2 (90% query coverage, 94.66% similarity) and to *Citrobacter* phage SH4 (84% query coverage, 94.44% similarity) (32, 33). Overall, the genome architecture and similarity to other bacteriophage genomes suggested that phage EP1 was similar to T7-like bacteriophages. The phage EP1 genome showed synteny with the *Shigella* phage SFPH2 genome. Except for two ORFs, all the other ORFs showed a similarity at the amino acid level to ORFs from SFPH2 (44.68% to 100% similarity). One predicted ORF (UNA02530.1) encoding a putative nucleotide kinase was absent from phage SFPH2 but showed high similarity to *Cronobacter* phage vB_CtuP_B1 (97% coverage, 93.53% identity) (34). Although UNA02530.1 was predicted to be a nucleotide kinase, it was still classified as a conserved phage protein with a domain of unknown function (DUF3310). The predicted ORF UNA02557.1, which is most likely a tail fiber/tail spike protein, showed no similarity to *Shigella* phage SFPH2, *Citrobacter* phage SH4, or any other closely related phage. The highest sequence similarity at the amino acid level was to Escherichia phage ZG49 (30% query coverage, 89.03% identity), with all of the matching residues present in the N-terminal region (35). These tail fiber proteins have been implicated in determining the host range and specificity of bacteriophages.

### Characterization of *Serratia* phage SP1.

*Serratia* phage SP1produced clear plaques of ~1 to 2 mm on its host *Serratia* sp., indicating a lytic life cycle. Phage SP1 had an icosahedral head ~90 nm long and ~70 nm wide, attached to a sheathed and contractile tail ~100 nm long (Fig. S1B). Based on the morphology of phage SP1, it was classified as a member of the *Myoviridae* family.

Phage SP1has a circularly permuted genome of 168,080 bp, with a 42.10% G+C content. The genome has 253 predicted protein coding ORFs and three tRNAs. Of the 253 ORFs, 144 (56.91%) were classified as hypothetical and could not be assigned a definitive function. Forty-three ORFs were predicted to be structural proteins, 39 were predicted to be nucleotide-related, and 4 were predicted to be lysis-related; the remaining 23 ORFs were assigned a function but could not be classified into the categories listed above (Fig. 1B).

Genome comparison of phage SP1 showed a high degree of similarity at the nucleotide level to *Serratia* phage Muldoon (98% query coverage, 91.08% similarity) and *Serratia* phage PS2 (84% query coverage, 85.33% similarity) (36, 37). The morphology, genome arrangement, and existence of other closely related bacteriophages suggested that *Serratia* phage SP1could be categorized as a T4-like phage. Most of the protein from SP1 (243/253) showed similarity to proteins from phage Muldoon. Four proteins showed no similarity to any known proteins in the database. Four ORFs showed similarity to Serratia phage PS2, one to *Serratia* phage 4S, and one to *Enterobacteria* phage RB27 (38, 39). Interestingly, phage SP1 had a predicted HNH endonuclease with similarity to a group I intron. This intron-like endonuclease is absent from other closely related *Serratia* phages. The closest match was present in Klebsiella phage vB_KaeM_KaAlpha, which also belongs to the Myoviridae family. Similar endonucleases were also present in multiple previously sequenced E. coli genomes.

### Characterization of *Elizabethkingia* phage EKP1.

*Elizabethkingia* phage EKP1 produced small <1mm turbid plaques on its host, which are typical of a lysogenic life cycle. Transmission electron microscopy showed that phage EKP1 had an icosahedral head with a length and width of ~50 nm and a contractile tail of ~110 nm (Fig. S1C). Based on its general morphological features, phage EKP1 was classified as a member of the *Myoviridae* family.

Phage EKP1 has a double-stranded DNA genome of 38,890 bp and a G+C content of 51.30%. The genome has 62 predicted protein coding ORFs and no predicted tRNAs. Most of the predicted ORFs from this phage were classified as hypothetical proteins or proteins containing domains of unknown function(s) (42/62, 67.74%). Seven ORFs were assigned as structural proteins, eight were predicted to have nucleotide-related functions, and four were predicted to have cell lysis-related functions. Among the nucleotide-related ORFs, a site-specific integrase and a tyrosine-type recombinase/integrase were predicted, confirming its lysogenic life cycle. Phage EKP1 also had a predicted ORF classified as a putative pyocin activator protein.

Comparative genome analysis of phage EKP1 showed very low nucleotide sequence similarity to known sequenced phage genomes (Klebsiella phage ST405-OXA48phi1.2, 10% query coverage and 73.41% identity; phage NG54, 4% query coverage, 75.20% identity) (40, 41). Interestingly, the EKP1 genome showed similarity to integrated prophage regions in the genome of bacteria of the *Metakosakonia* sp. MRY16-398 (22% query coverage, 95.98% identity) (42) and *Enterobacteriaceae* bacterium ENNIH3 (51% query coverage, 86.93% identity). At the protein level, most of the ORFs from EKP1 showed similarity to various proteins from partial genomes of bacteriophages sequenced from the human gut (43). The genome of EKP1 showed loose synteny to phage NG54, which was assembled from viromes of *Nasonia* wasps (Fig. 1C) (41). Phage EKP1 showed no similarity to the previously reported *Elizabethkingia* phage TCUEAP (44).

### Host range of bacteriophages.

Host specificity determined by spot assays and plaque assays confirmed that bacteriophages EP1, SP1, EKP1, and GH1 did not lyse or infect any of the bacteria tested other than their original host (Table S1). These results revealed that the bacteriophages used in the study are highly specific for their particular hosts and do not affect other bacteria selected for the study.

10.1128/mbio.03017-22.5 TABLE S1 Host specificity of bacteriophages used in the study. +, lysis in spot assay test; -, no lysis in spot assay test; P, plaques in double-layer agar test; N, no plaques in double-layer agar test. Download Table S1, DOCX file, 0.01 MB. Copyright © 2022 Tikhe and Dimopoulos. 2022 Tikhe and Dimopoulos. https://creativecommons.org/licenses/by/4.0/ This content is distributed under the terms of the Creative Commons Attribution 4.0 International license.

### Bacterial growth assay.

Bacterial growth curve assays showed that addition of the appropriate bacteriophages could suppress the growth of their respective hosts. Enterobacter phage EP1, *Serratia* phage SP1, and Pseudomonas phage GH1 completely suppressed the growth of their respective host bacteria for 8 h, with the OD_600_ remaining close to zero (Fig. S2A to S2C). Interestingly, after 24 h, most of the bacterial cultures with added bacteriophages showed a significant increase in the OD_600_ compared to the 8-h value. However, this increase was still significantly lower than the 24-h OD_600_ value of their respective controls. Unlike the bacteriophages, addition of *Elizabethkingia* phage EKP1 had little to no effect on the growth kinetics of its host, as there was no significance difference between the OD_600_ values at any of the time points tested (Fig. S2D).

### Gnotobiotic *An. gambiae* larval development assays.

First, we set up *An. gambiae* gnotobiotic larval survival assays with individual bacteria and their respective phages. Since we were unable to isolate bacteriophage against *Asaia*, larval survival assays were carried out with the bacteria alone.

**FIG 2:**
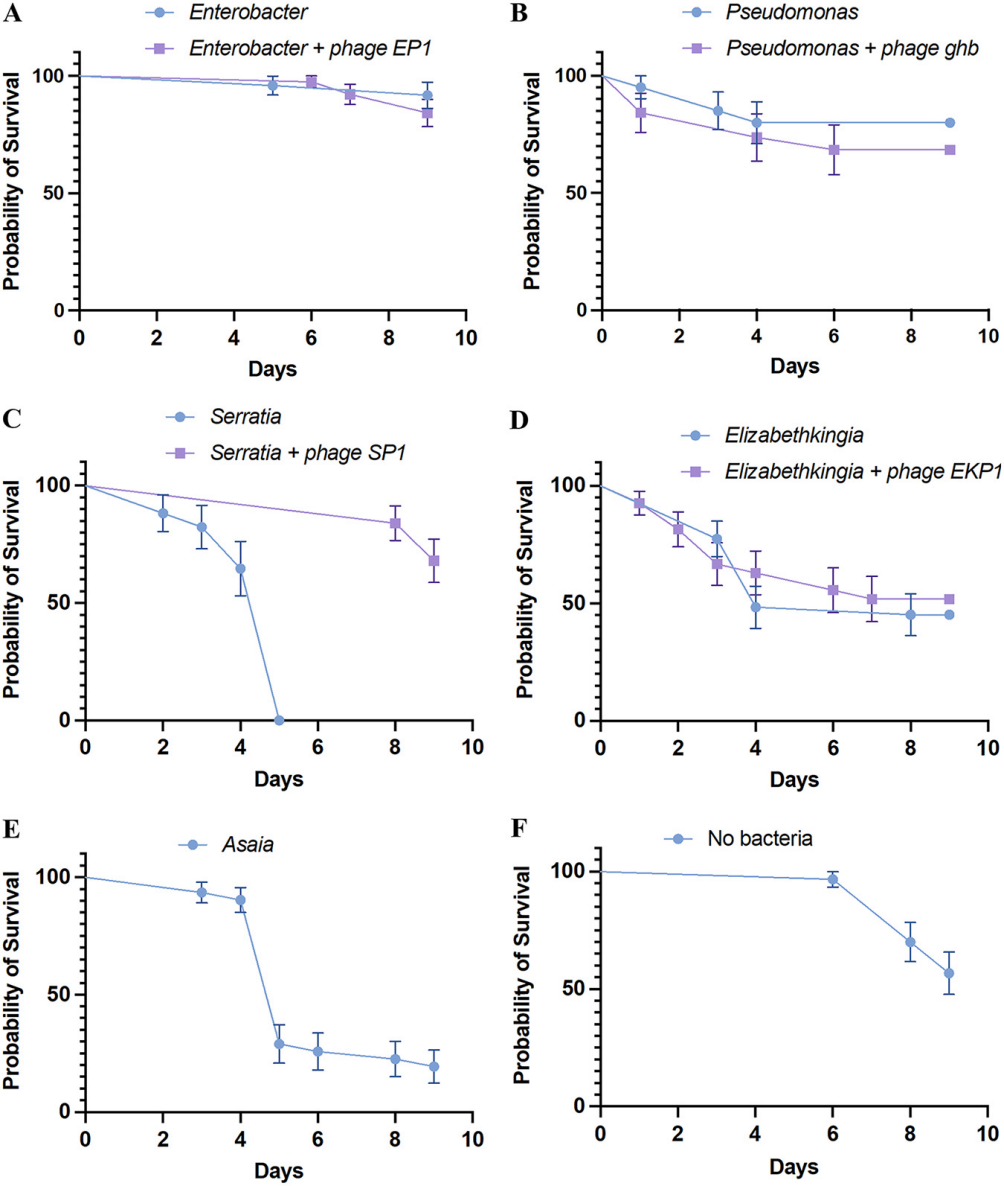
Kaplan-Myer survival probabilities of *An. gambiae* larvae in a gnotobiotic setting with individual bacteria and their respective phages. (A) Enterobacter, Enterobacter + phage EP1; (B) Pseudomonas, Pseudomonas + phage GH1; (C) *Serratia*, *Serratia* + phage SP1; (D) *Elizabethkingia*, *Elizabethkingia* + phage EKP1; (E) *Asaia*. (F) No bacteria. Except for *Serratia*, there was no significant difference in the survival proportions of larvae with the addition of bacteriophages. Error bars represent standard error of the mean (SEM).

**FIG 3:**
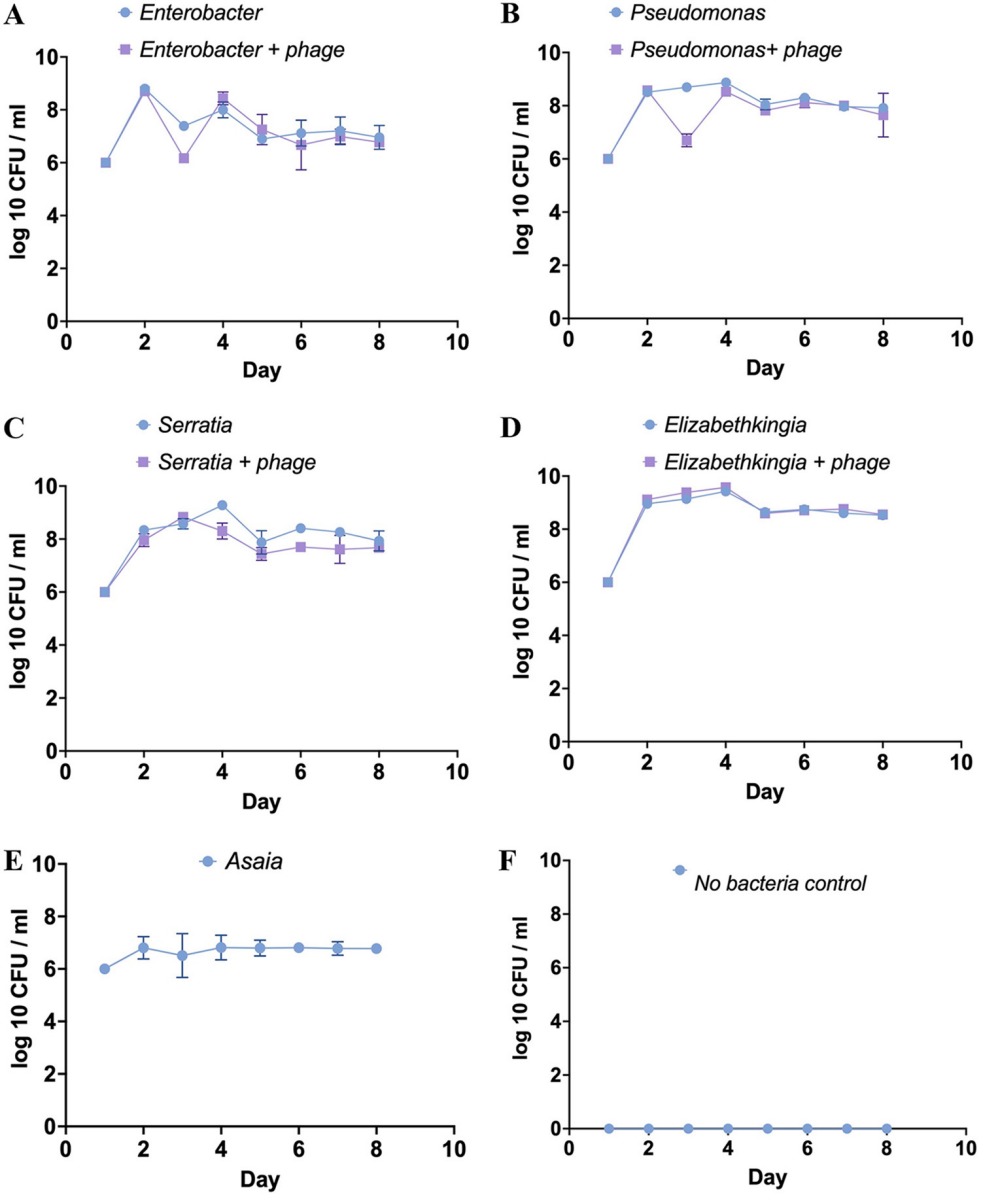
Bacterial numbers (log_10_) per mL of larval water habitat measured every day with and without the addition of the respective bacteriophages. (A) Enterobacter, Enterobacter + phage EP1; (B) Pseudomonas, Pseudomonas + phage GH1; (C) *Serratia*, *Serratia* + phage SP1; (D) *Elizabethkingia*, *Elizabethkingia* + phage EKP1; (E) *Asaia;* (F) no bacteria control. Error bars represent SEM.

For Enterobacter gnotobiotic larvae, the average survival was 91.3% after 9 days. When Enterobacter phage EP1 was added to the water, the larval survival was 84.21%. There was no statistical difference in the survival probabilities of Enterobacter gnotobiotic larvae with and without exposure to phage EP1 (log-rank [Mantel-Cox] test, *P* = 0.4107; Fig. 2A). After the addition of bacteriophage EP1, there was a significant reduction in the Enterobacter concentration (1.47 × 10^6/^mL in the water the next day), compared to the Enterobacter control (mean, 2.45 × 10^7^/mL; Fig. 3A). However, there was no difference in the bacterial concentration in the water between Enterobacter and Enterobacter phage EP1 treatment from day 4 to day 9 (Fig. 3A). There was also no significant difference in the pupation percentage after 10 days between Enterobacter (mean pupation, 86.66%) and Enterobacter phage EP1 treatment (mean pupation, 93.33%; Fig. S3).

10.1128/mbio.03017-22.2 FIG S2 Growth curves of bacteria with and without the addition of their respective phages. (A) Enterobacter, Enterobacter + phage EP1; (B) Pseudomonas, Pseudomonas + phage GH1; (C) *Serratia*, *Serratia* + phage SP1; (D) *Elizabethkingia*, *Elizabethkingia* + phage EKP1. Download FIG S2, TIF file, 2.0 MB. Copyright © 2022 Tikhe and Dimopoulos. 2022 Tikhe and Dimopoulos. https://creativecommons.org/licenses/by/4.0/ This content is distributed under the terms of the Creative Commons Attribution 4.0 International license.

10.1128/mbio.03017-22.3 FIG S3 Pupation (percentage) of *An. gambiae* larvae after 10 days. Larvae were grown in a gnotobiotic setting with Enterobacter, Pseudomonas, *Serratia*, *Elizabethkingia*, and *Asaia* and with the addition of their respective phages EP1, GH1, SP1, and EKP1. Download FIG S3, TIF file, 0.8 MB. Copyright © 2022 Tikhe and Dimopoulos. 2022 Tikhe and Dimopoulos. https://creativecommons.org/licenses/by/4.0/ This content is distributed under the terms of the Creative Commons Attribution 4.0 International license.

Gnotobiotic *An. gambiae* grown with Pseudomonas putida alone and those treated with phage GH1 did not show any difference in survival probability over the course of 9 days (final average larval survival after 9 days: *Ps. putida *= 80%, *Ps. putida* with phage GH1 = 68.42%, log-rank [Mantel-Cox] test, *P* = 0.428; Fig. 2B). However, there was a significant difference in the percent pupation between the treatments (ANOVA, *P* = 0.0038; Fig. S3). Gnotobiotic larvae with *Ps. putida* alone had higher percent pupation after 10 days (average pupation, 73.33%) than did those exposed to phage GH1 (average pupation, 53.33%). As in the case of Enterobacter, addition of phage GH1 reduced the Pseudomonas numbers in the water on the next day compared to the respective control (*t* test, *P* = 0.000168 on day 3; Fig. 3B). Except for day 3 (1 day after adding the phage), there was no significant difference in the bacterial numbers between the Pseudomonas and phage GH1 treatments.

The *Elizabethkingia* gnotobiotic larvae showed lower survival probability than did the Enterobacter or Pseudomonas gnotobiotic larvae (log-rank [Mantel-Cox] test, Enterobacter versus *Elizabethkingia*, *P* = 0.0003; Pseudomonas versus *Elizabethkingia*, *P* = 0.0279). There was no difference in the survival probability of larvae between *Elizabethkingia* alone and with the addition of phage EKP1 (survival after 9 days: *Elizabethkingia*, 45.16%; *Elizabethkingia* + phage EKP1, 51.85%; Fig. 2D). Addition of phage EKP1 did not have an effect on *Elizabethkingia* density in the water on any of the days (Fig. 3D).

*An. gambiae* larvae grown with *Serratia* alone showed very high mortality, with all the larvae having died by day 5. However, when *Serratia* phage SP1 was added to the water, the larval survival improved dramatically, to an average of 68% by day 9 (log-rank [Mantel-Cox] test, *P* < 0.0001; Fig. 2C). Interestingly, the *Serratia* gnotobiotic larvae never developed past the first instar. Even though the addition of phage SP1 improved the larval survival, it had no effect on larval development, as all the larvae failed to develop past the first instar during the 9 days. The *Serratia* concentration was significantly lower the next day after addition of phage SP1 compared to the control value (*Serratia* only, *t* test, *P* = 0.005215; Fig. 3C). Since all the larvae receiving the *Serratia* treatment died by day 5, bacterial numbers in the water were measured after removing all the dead larvae after day 5.

Similarly, the *Asaia* gnotobiotic larvae showed high mortality, with about 80% mortality by day 9 (Fig. 2E). Also, all the larvae grown with *Asaia* did not develop past the first instar during the course of the experiment (Fig. S3). Since we were unable to isolate any bacteriophage infecting *Asaia*, gnotobiotic experiments were carried out only with *Asaia*, without the addition of any bacteriophage (Fig. S3E).

*An. gambiae* larvae grown without the addition of any bacteria remained as first instars throughout the course of the experiment, and no bacterial contamination was detected during the experiment (Fig. S3F). Larvae without any bacteria showed a mean survival probability of 56.66 at the end of the experiment (Fig. 2F).

### Gnotobiotic *An. gambiae* larval development assays with a defined bacterial community.

In the next assay, we analyzed *An. gambiae* larval survival and development in a gnotobiotic-defined bacterial community composed of all the five bacteria tested above, namely, Enterobacter, Pseudomonas, *Serratia*, *Elizabethkingia*, and *Asaia*. Gnotobiotic larvae with a community of all five bacteria were used as the control.

**FIG 4:**
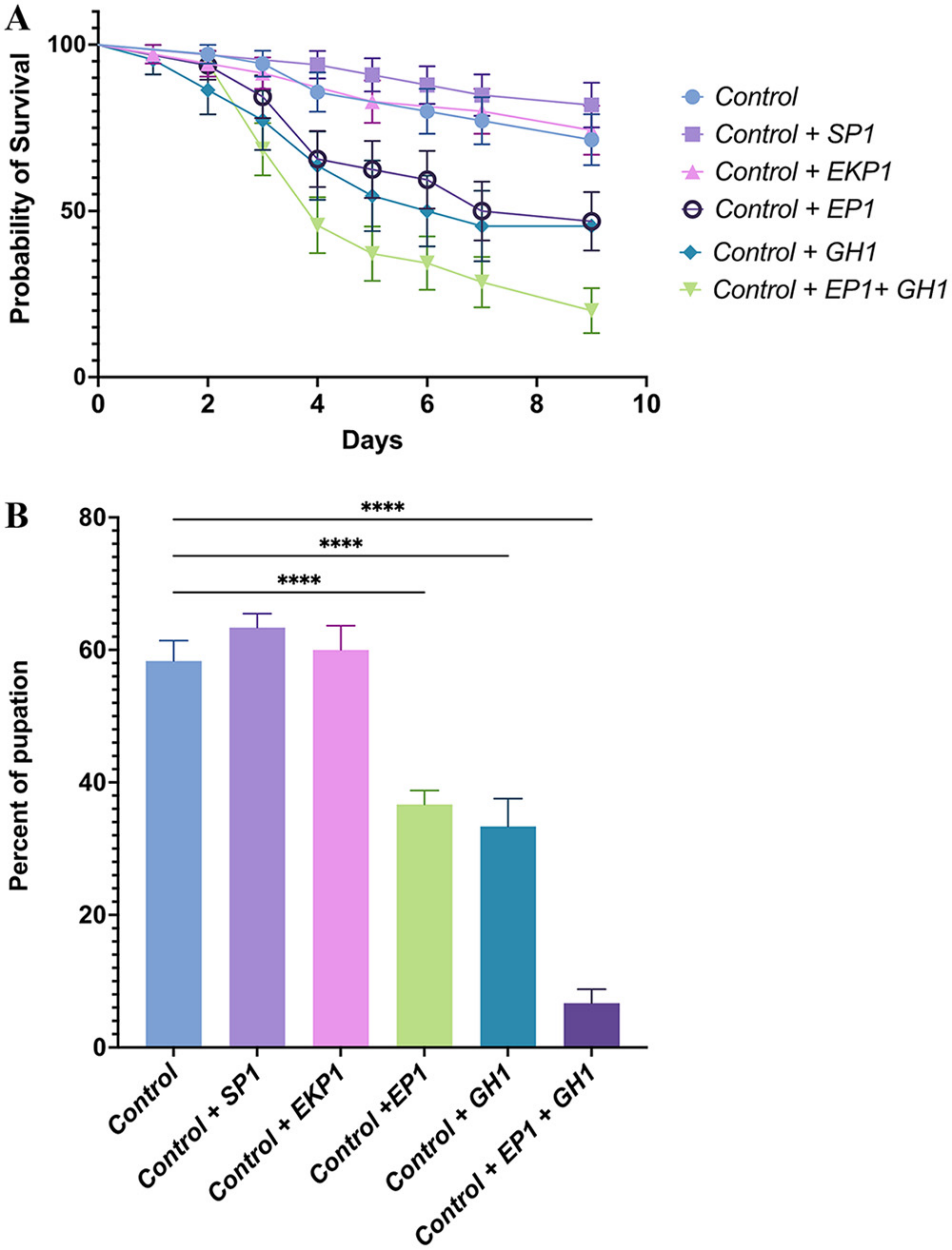
(A) Kaplan-Myer survival probabilities of *An. gambiae* larvae. (B) Pupation percentage of A. gambiae larvae after 10 days. Larvae were grown in a gnotobiotic setting with a known bacterial community of Enterobacter, Pseudomonas, *Serratia*, *Elizabethkingia*, and *Asaia* (control), with individual phages SP1, EKP1, EP1, and GH1, and with EP1 and GH1 together.

*An. gambiae* larvae growing under gnotobiotic growth conditions with five bacteria (control) had a mean survival rate of 71.28% after 9 days (Fig. 4). There was no significant difference in the larval survival rate between the control and the phage SP1 or phage EKP1 treatments. Phage SP1 treatment yielded slightly higher survival rate; however, this increase was not statistically significant. With the addition of Enterobacter phage EP1, the larval survival significantly decreased to 46.87% compared to the control (log-rank [Mantel-Cox] test, *P* = 0.0302). Similarly, the addition of Pseudomonas phage GH1 significantly reduced the larval survival to 47.82% compared to the control (log-rank [Mantel-Cox] test, *P* = 0.0240). When both the EP1 and GH1 bacteriophages were added together, the larvae had the lowest survival probability, with a mean survival of 20% after 9 days (log-rank [Mantel-Cox] test, *P* = 0.0002 compared to the control, Fig. 4A). The mean pupation rates (at 10 days) after the addition of EP1 (36.67%) and GH1 (33.33%) separately and together (6.33%) were also significantly reduced compared to the control (58.33%, ANOVA with Tukey’s multiple-comparison test, *P* < 0.0004; Fig. 4B). There was no significant difference in the pupation rates between the control *Serratia* phage SP1 and *Elizabethkingia* phage EKP1 treatment (63.33% and 60%, respectively, *P* > 0.5).

There was no difference in the bacterial number in the water between control and any of the treatments on all the days (*P* > 0.8, ANOVA with Dunnett's multiple-comparison test; Fig. S4)

10.1128/mbio.03017-22.4 FIG S4 Bacterial numbers (log10) per mL of larval water habitat measured every day with and without the addition of the respective bacteriophages. Control: bacterial community comprising Enterobacter, Pseudomonas, *Serratia*, *Elizabethkingia* and *Asaia*. Individual bacteriophages were added to the water on day 2: Enterobacter phage EP1, Pseudomonas phage GH1, *Serratia* phage SP1, *Elizabethkingia* phage EKP1, and Enterobacter phage EP1 and Pseudomonas phage GH1 added together. Download FIG S4, TIF file, 0.2 MB. Copyright © 2022 Tikhe and Dimopoulos. 2022 Tikhe and Dimopoulos. https://creativecommons.org/licenses/by/4.0/ This content is distributed under the terms of the Creative Commons Attribution 4.0 International license.

## DISCUSSION

As underscored here, the mosquito-associated microbiota plays a critical role in mosquito biology. Despite the importance of the mosquito microbiota, interactions within the microbiota and their applied utility for altering mosquito biology have been essentially unstudied. The data presented here demonstrate that bacteriophages can be utilized as an ideal tool for mosquito microbiota research. We have now isolated and characterized novel bacteriophages infecting bacteria commonly associated with the malaria vector mosquito *An. gambiae*. We evaluated the utility of these bacteriophages for specifically targeting the mosquito’s host bacteria and have characterized their effects on mosquito biology as a proof of principle for the use of such interactions in mosquito control. In particular, we have now studied mosquito larval survival and development as a phenotype for evaluating the effects of bacteriophage-mediated targeted reduction in the numbers of particular bacteria. Our results indicate that adding bacteriophages to water against bacteria that support larval devolvement and survival does indeed have a negative impact on the fitness of mosquito larvae. Thus, our study validates the utility of bacteriophages for mosquito microbiota research.

Given the immense importance of *An. gambiae* as a principal vector of Plasmodium parasites on the African continent, we performed our experiments in this mosquito species. The mosquito microbiota is complex and diverse and varies according to geographical location, mosquito species, and environmental conditions (9, 19, 21, 22, 45). Despite this variability, however, certain bacterial genera are found be more frequently associated with mosquitoes. Enterobacter, Pseudomonas, *Serratia*, *Elizabethkingia*, and *Asaia* have been isolated from multiple species of lab mosquitoes as well as field-collected adults and larvae (23, 26). These five bacterial genera have been hypothesized to be part of the core microbiota of *An. gambiae* mosquitoes (26). For this reason, we chose these five bacterial genera for our study.

As a first step, we isolated bacteriophages against representative members of the five aforementioned bacterial genera. Except for Pseudomonas, all the bacterial isolates used in the study were isolated from *An. gambiae* mosquitoes from our lab colony. In order to increase our chances of isolating bacteriophages, we utilized sewage water samples from a wastewater treatment plant, since sewage water is a rich source of bacteriophages. We were able to isolate bacteriophages against all the bacteria except *Asaia*. This was not surprising, since *Asaia* is an acetic acid bacterium, and sewage water is an unlikely source of bacteriophages against this genus. There are very few reports in the literature of bacteriophages infecting acetic acid bacteria. We also attempted to utilize fermented fruits, live culture kombucha, and local flower nectars as sources, but were unable to isolate any *Asaia* bacteriophage. We tested the only known bacteriophage available in the phage bank, phage Werquin, which infects *Gluconobacter*, against *Asaia*; however, it was unable to infect *Asaia*. For Pseudomonas isolated from *An. gambiae*, we were able to isolate bacteriophages, but surprisingly we were unable to maintain these bacteriophages as stocks. Instead, we utilized a previously well-characterized phage, GH1, which infects *Ps. putida* (ATCC 12633). The lab isolate of Pseudomonas supported *An. gambiae* larval development (data not shown). *Ps. putida* ATCC 12633 also supported normal *An. gambiae* larval development, and no significant mortality was observed. Because of its similar phenotypes with regard to *Anopheles* larval development, we were able to utilize *Ps. putida* ATCC 12633 and its phage, GH1. Phage GH1 is a highly lytic T7-like bacteriophage whose genome is already sequenced. Thus, we successfully isolated three novel bacteriophages, designated EP1, SP1, and EKP1, infecting Enterobacter, *Serratia*, and *Elizabethkingia*, respectively.

For microbiome modulation, targeted lytic phages are desired. Enterobacter phage EP1 and *Serratia* phage SP1 proved to be highly lytic and host-specific phages, which made them ideal candidates. Enterobacter EP1 was a T7-like bacteriophage. Multiple T7-like bacteriophages infecting a diverse range of bacterial genera have been isolated and characterized (46 to 48). Phage EP1 was closely related to *Shigella* phage SFPH2 (32), although it showed differences in the tail fiber proteins; these tail proteins have been known to determine the host range of the phage (49, 50). A previous study showed that switching the tail proteins between bacteriophages can alter the host range of T7-like bacteriophages (49). Phage EP1 did not infect any of the other bacteria tested. However, if needed, phage EP1 can be further evaluated and engineered to alter its range; T7-like bacteriophages have already been evaluated extensively for *in vivo* phage therapy (51, 52). Engineered T7 phages have been utilized as biotechnological tools because of their ability to express foreign peptides on their capsids (53). T7-like phages have been extensively studied with respect to their biology and also with regard to applied uses, making them ideal candidates for phage therapy (54).

*Serratia* phage SP1 proved to be a T4-like phage. It showed a high degree of synteny to *Serratia* phage Muldoon, another T4-like phage (37). The most important difference between these two phages was the presence of an intron-like endonuclease in phage SP1. These intron-like endonucleases are widespread in T4-like phage genomes, but their role in phage biology remains elusive (55). Like T7 phages, T4-like phages have been isolated from various environments in which they infect a variety of bacteria (56, 57). T4-like phages have also been evaluated for phage therapy and for their biotechnological potential (52, 58).

Unlike phages EP1 and SP1, *Elizabethkingia* phage EKP1 was lysogenic in nature. Lysogenic phages are not ideal candidates for microbiome engineering or phage therapy. Phage EKP1 showed no clear homology to a previously isolated bacteriophage. Only one *Elizabethkingia* phage is available in the database, but it did not show any similarity to phage EKP1 (44). Overall, there is a dearth of characterized bacteriophages infecting *Bacteroidetes*. Phage EKP1 showed multiple matches to uncultured human virome phages (43). Phage EKP1 can be fully characterized at better resolution when the full genomes of the matching bacteriophages from the human gut virome become available.

*In vitro* bacterial growth curves in the presence of phage EP1, SP1, or GH1 showed a high degree of bacterial suppression for 8 h. However, after 24 h, the bacterial growth (monitored by OD_600_) increased. This result points to the possibility of the emergence of bacteriophage resistance in the culture, inactivation of the phages, or phage–bacteria homeostasis. Emergence of phage resistance has been well documented *in vivo* during phage therapy and in *in vitro* lab-based experiments (59, 60). Irrespective of the reason, these data indicate that complete suppression and lysis of the host bacteria with a single phage might be challenging. For this proof-of-principle study, we utilized a series of bacteriophages, but each one targeted only one specific bacterial genus. However, utilizing a cocktail of diverse phages may reduce the emergence of phage resistance. It has been shown that phage resistance can be developed with a single phage, but not with a combination of multiple phages (60).

*Elizabethkingia* phage EKP1 had no effect on the growth kinetics of its host. Phage EKP1 was lysogenic and also formed small turbid plaques on its host. The growth curve analysis showed that phage EKP1 may not be an ideal candidate for reducing *Elizabethkingia* numbers in culture. Lysogenic bacteriophages can be converted to lytic bacteria by removing their lysogenic machinery (61). In recent years, protocols have been established to remove or replace genes from phage genomes. An engineered lysogenic bacteriophage has already been successfully used in phage therapy to treat a patient suffering from a chronic infection with antibiotic-resistant bacteria (62). Thus, if required, *Elizabethkingia* phage EKP1 has the potential to be utilized for phage therapy.

For successful modulation of the microbiota in any system, the host specificity of the bacteriophages is key. Bacteriophages can have a very narrow host range and can be strain specific; however, wide host-range phages have also been reported. It is important to note that certain bacteriophage can lyse nonhost bacteria without actually infecting them. This phenomenon can reduce the number of nontarget bacteria and improve target-specific microbiome modulation. Hence, it is important to study the host range of phages with a spot test as well as plaque assays. Spot tests can only reveal a localized lysis of cells and do not accurately indicate infection or replication of phages. However, the standard double-agar method will differentiate between phage infection and lysis. Our host range analysis showed that all the phages used in the study were specific as they did not infect or lyse other bacteria used in the study. This specificity made these phages ideal for targeted removal of specific desired bacteria from a defined bacterial community comprising all five bacteria genera.

In order to understand the impact of different bacteria in water on *Anopheles* larval development, we set up larval development assays in a controlled gnotobiotic environment with individual bacteria added separately to the water. We also determined whether the addition of a single bacteriophage in this setting had any effect on larval development. To the best of our knowledge, this is the first study of *Anopheles* mosquitoes in a gnotobiotic setting. Our results clearly showed that the type of bacteria present in the water has a very strong effect on *Anopheles* larval development. Of the five bacteria tested, Enterobacter and Pseudomonas showed little effect on larval development, with no noticeable mortality resulting from treatment. Most of the larvae with Enterobacter or Pseudomonas pupated successfully. Even though the addition of phages EP1 and GH1, respectively, reduced Enterobacter and Pseudomonas numbers in the water for one at least a day, they had no significant impact on larval development. Despite the fact that a lower bacterial density in the water can delay larval development, the reduction observed in response to phage treatment may not be sufficient to have a significant impact (12, 13).

*Elizabethkingia* gnotobiotic larvae had a lower survival rate and pupation success than did those of Enterobacter and Pseudomonas. *Elizabethkingia* has been reported to colonize multiple species of *Anopheles* larvae and is also capable of vertical, horizontal, and transstadial transmission (63 to 66). However, in a gnotobiotic setting, *Elizabethkingia* alone may be pathogenic to the larvae or may compete for essential nutrients with the larvae. Compared to the other bacteria, *Elizabethkingia* showed a higher density per mL of water, which may have caused changes in the physical and biochemical properties of the water and thereby resulted in higher mortality and lower pupation rates. Addition of phage EKP1 did not result in improved larval survival or pupation rates; however, this result was not unexpected because phage EKP1 is lysogenic and did not have an effect on bacterial growth during *in vitro* assays.

*Serratia* has been reported to be a pathogen as well as a symbiont of mosquitoes (67 to 69). However, there are no studies analyzing the effect of *Serratia* on larval development in a gnotobiotic setting. *An. gambiae* larvae grown with only *Serratia* in the water had a very high mortality, with all the larvae dying by day 5. None of the larvae with *Serratia* developed past the first instar. Interestingly, with the addition of phage SP1 we saw the most dramatic phenotype, with extremely improved larval survival compared to *Serratia* alone. Addition of phage SP1 reduced the *Serratia* numbers the next day. Despite massive improvement in the larval survival, addition of phage SP1 did not rescue larval development. This result suggests that *Serratia* is unable to support larval development. The improved larval survival could be explained by the trade-offs resulting from the development of phage resistance. As previously seen in the *in vitro* experiments, an increase in *Serratia* growth was observed 24 h after the beginning of the phage treatment, suggesting the development of phage-resistant mutants in the population. In a previous study, Serratia marcescens phage-resistant mutants were less virulent to *Drosophila* and were also easily targeted by the *Cecropia* immune system compared to the parent strain (70). A study of hypervirulent Klebsiella pneumoniae has shown a dramatic decrease in virulence in phage-resistant strains in a moth model (71). Multiple studies have demonstrated significant physiological changes in bacterial hosts after the development of phage resistance. It would be interesting to further analyze the effect of phage-resistant *Serratia* isolates on mosquito larval development and pathogenicity in future studies.

A previous study has shown that *Asaia* stably colonizes the larval gut of *An. stephensi*, and multiple studies have demonstrated the importance of *Asaia* for mosquito larval development (72 to 74). However, our results for gnotobiotic *An. gambiae* larvae with *Asaia* showed high larval mortality and no larval development past the first instar. *Asaia* numbers remained stable throughout the course of the experiment.

Recent studies have shown that the microbiota may supply the essential nutrient riboflavin, which is essential for larval development (11). There is a possibility that *Serratia* and *Asaia* do not provide larvae with riboflavin. It has been shown that not all bacteria can support larval development in a gnotobiotic setting. Initially, it was proposed that these bacteria may not colonize the larval gut, resulting in stalled larval development. However, in light of recent findings, this phenomenon needs to be revisited. It should be noted that the larval phenotypes observed in the study may be strain-specific as well as diet- and experimental design-specific. Different strains from the same species with different diets and experimental settings may have different effects on larval development in various mosquito species. Thus far, all the gnotobiotic studies have been performed in *Aedes* and *Culex* mosquitoes. As far as we are aware, our study is the first to be carried out in *An. gambiae* mosquitoes, the principal vector of *Plasmodium* parasites in Africa. Our results corroborate observations from other mosquitoes that have shown that not all bacteria support larval development and that the type of bacteria present in the water has a significant impact on larval survival and development.

Next, we decided to study larval survival and development by adding individual phages to a defined bacterial community comprising one of five bacteria or a combination of all five. *Elizabethkingia*, *Serratia*, and *Asaia* gnotobiotic larvae showed high mortality and low pupation rates. Noticeably, the adverse effects seen with gnotobiotic larvae of *Serratia*, *Asaia*, and *Elizabethkingia* were not observed when these bacteria were a part of a mixed bacterial community. There are multiple plausible reasons for this. (i) The bacterial number of individual isolates was lower than in the gnotobiotic setting. Many bacterial virulence genes are dependent on quorum-sensing, and individual bacteria do not reach high enough numbers to express virulence factors (75, 76). (ii) Enterobacter and Pseudomonas present in the community provide essential nutrients like riboflavin to larvae (11), which helps their development, making their immune system more competent and resulting in lower mortality.

We did not observe any significant difference in the larval survival between the control (gnotobiotic larvae with all five bacteria), phage SP1, and EKP1 treatment. This was not unexpected, because phage EKP1 did not have any effect on the bacterial numbers *in vitro* or *in vivo*, and *Serratia* phage SP1 had a positive effect on larval survival in a gnotobiotic setting. Based on the previous results, Enterobacter and Pseudomonas gnotobiotic larvae had the highest survival and pupation rates. Interestingly, adding phages EP1 and GH1 either separately or together negatively affected larval survival. These results were interesting because these bacteriophages had no effect on larval development or survival in a gnotobiotic setting. These data suggest that the addition of phage EP1 and/or GH1 reduces Enterobacter and Pseudomonas numbers in the water, which most likely increases *Serratia*, *Elizabethkingia*, and *Asaia* numbers, resulting in increased larval mortality.

One of the limitations of our study is that we did not examine changes in the bacterial community composition in the water by 16s sequencing after each phage treatment. As the total number of bacteria in the water remained the same following phage treatment, microbial dysbiosis caused by bacteriophage that resulted in an increased larval mortality is the most likely explanation for our results. Bacterial dysbiosis has been shown to accelerate mortality in *Anopheles* mosquitoes (77). Along with mosquitoes, bacterial dysbiosis affects multiple biological processes in other insects and mammals (78 to 81). In the mouse model, phage-induced dysbiosis has been shown to have an impact on the metabolite profile in the gut (31). Resident prophages have been proposed as causative agents of gut dysbiosis, which is known to be responsible for multiple disorders in humans (82).

Overall, we have shown here that bacteriophages have the potential to play a key role in mosquito microbiota research. Based on larval development assays, targeted phage therapy for larval control is feasible. Phages can be combined with insect growth regulators or other larvicidal agent such as B. thuringiensis subsp. *israelensis*. Although this was a proof-of-principle study and therefore limited in scope, it nevertheless suggests that a large-scale study to obtain a detailed understanding of local bacteria and their respective phages is clearly warranted. Here, we only looked only at larval development as a phenotype, but it is very likely that phage treatment affects other phenotypes in adult mosquitoes, such as vector competence and reproduction. Engineered bacteriophages infecting resident gut bacteria also have the potential to be used as paratransgenesis agents. Most of the bacteria isolated from mosquitoes are opportunistic human and animal pathogens and resistant to multiple antibiotics, and these phages can be utilized in phage therapy to treat infections with any bacteria.

## MATERIALS AND METHODS

### Bacteriophage isolation.

Pretreatment sewage water was collected from the Back River wastewater treatment plant, Baltimore, Maryland. The water was centrifuged at 5,000 rpm for 10 min, and the supernatant was filtered through a 0.22-μm vacuum filter. This filtered supernatant was used to isolate bacteriophages against Enterobacter, Pseudomonas, *Serratia*, *Elizabethkingia*, and *Asaia* using a standard double-layer bacteriophage isolation method. All the bacterial strains were previously isolated from the lab colony of *An. gambiae* mosquitoes from the Johns Hopkins Malaria Research Institute insectary, Baltimore, Maryland, United States. Individual plaques were purified from the plates, and after multiple passages, high-titer lysates of pure bacteriophages were prepared using a phage on tap protocol (83). Bacteriophages were stored at 4°C in phage buffer until used.

Out of the five bacteria selected, we were able to isolate and maintain bacteriophages infecting Enterobacter, *Serratia*, and *Elizabethkingia*. For Pseudomonas, we utilized a previously well-characterized bacteriophage, GH1. We were unable to isolate bacteriophage infecting *Asaia*. We tested a known phage *Gluconobacter* phage Werquin from The Félix d'Hérelle Reference Center for Bacterial Viruses (HER201) against *Asaia*, but we found that it does not infect *Asaia*.

### Bacteriophage host range and morphology.

The phage host range was determined by spot assay tests and plaque assay tests using the double-layer agar method. Five selected bacteria, namely, Enterobacter sp., *Serratia* sp., *Elizabethkingia* sp., Pseudomonas putida., and *Asaia* sp., were used. For the spot assay tests, purified cultures of bacteriophages were serially diluted in phage buffer, and 10 μL of phage solution was spot- inoculated onto a lawn of five selected bacterial strains and the zone of lysis observed. For the double-layer agar assays, bacteriophage dilutions were mixed with all five bacteria individually and observed for the presence of plaques.

A drop of purified phage culture was applied to a copper grid and stained with 2% uranyl acetate for electron microscopy. Phage morphology was observed under a Hitachi 7600 TEM electron microscope at the Johns Hopkins MicFac facility.

### Bacteriophage genome sequencing.

Bacteriophage DNA was extracted using a previously described protocol. *De novo* phage genomes were sequenced at MR DNA (Molecular Research LP), using Illumina MiSeq paired-end sequencing. De novo bacteriophage assembly and annotation were done according to a previously described protocol (84). Bacteriophages were classified using VIRFAM analysis (85). Bacteriophages infecting Enterobacter were designated EP1, bacteriophages infecting *Serratia* were designated SP1, and bacteriophages infecting *Elizabethkingia* were designated EKP1.

### Bacterial growth curves.

Cultures of Enterobacter sp., *Serratia* sp., Pseudomonas putida, and *Elizabethkingia* sp. were diluted to an OD_600_ of 0.1 in 25 mL LB broth in a 50-mL Falcon tube. The respective purified bacteriophage cultures in phage buffer were added to the medium at an approximate multiplicity of infection (MOI) of 0.1. Control bacterial cultures were supplemented with the same volume of phage buffer. The OD_600_ was measured every hour for 8 h and after 24 h. The OD_600_ values for the control cultures (without added bacteriophage) and phage treatment cultures were compared.

### Generation of axenic mosquito larvae.

*An. gambiae* Keele strain mosquitoes were used for all experiments. Gnotobiotic mosquito larvae were generated as described in previous protocols (8, 9). In brief, *An. gambiae* eggs were collected on filter paper, and eggs were surface-sterilized with 70% ethanol for 5 min, followed by sterilization with 3% bleach and 0.1% D-256 disinfectant (Vedco) for 5 min, and another wash with 70% ethanol for 5 min. Eggs were washed twice with sterile distilled water and then allowed to hatch in sterile distilled water in a cell culture flask sealed with Parafilm for 48 h at 27°C in a 12-h light–dark cycle incubator with 80% relative humidity. All these processes were carried out in a sterile biosafety cabinet.

### Gnotobiotic larval rearing.

Once the eggs hatched, 10 L1 larvae were transferred to a well in a six-well plate containing 5 mL of water. Each well received 3.5 mg of a sterilized diet (tropical fish flakes: rabbit chow: liver powder, 1:1:2) on days 1, 3, 5, and 7. On day 1, 1 × 10^6^ per mL of each of the five bacteria (Enterobacter, Pseudomonas, *Serratia*, *Elizabethkingia*, and *Asia*) was separately added to the water. Larvae without any added bacteria served as a negative control. For the second experiment, an equal mixture of all five bacteria was used, with a final bacterial concentration of 1 × 10^6^/mL. For phage treatment, 1 × 10^6^/mL of each respective bacteriophage was added to the breeding water on day 2. For example, gnotobiotic larvae inoculated with Enterobacter were treated with phage EP1 on day 2. For the second experiment, 1 × 10^6^ per mL of Enterobacter phage EP1, Pseudomonas phage GH1, or an equal mix of both EP1 and GH1 was added to the water on day 2. Larval mortality and development were monitored every day for 9 days. Each day, 100 μL of water was removed from each well and serially diluted, then plated on lysogeny agar plates to measure bacterial numbers as previously described (86). All the experiments were repeated three times independently.

### Data availability.

The whole annotated genomes of *Enterobacter* phage EP1, *Serratia* phage SP1, and *Elizabethkingia* phage EKP1 are publicly available at GenBank under accession numbers OM457002.1, OM457001.1, and OM284015.1, respectively.
