# Supplementary material for: Phage Therapy for Mosquito Larval Control: a Proof-of-Principle Study
Source: mBio. 2022 Nov 29;13(6):e03017-22. doi: 10.1128/mbio.03017-22 (PMC9765668; doi:10.1128/mbio.03017-22)
Supplement: TABLE S1 [file mbio.03017-22-s0005.docx]

Supplementary Table 1

| Host / Phage | *Enterobacter* | *Serratia* | *Pseudomonas* | *Elizabethkingia* | *Asaia* |
| --- | --- | --- | --- | --- | --- |
| EP1 | +/ P | -/N | -/N | -/N | -/N |
| SP1 | -/N | +/ P | -/N | -/N | -/N |
| GH1 | -/N | -/N | +/ P | -/N | -/N |
| EKP1 | -/N | -/N | -/N | +/ P | -/N |
