# Supplementary figures and images for: Phage Therapy for Mosquito Larval Control: a Proof-of-Principle Study
Source: mBio. 2022 Nov 29;13(6):e03017-22. doi: 10.1128/mbio.03017-22 (PMC9765668; doi:10.1128/mbio.03017-22)

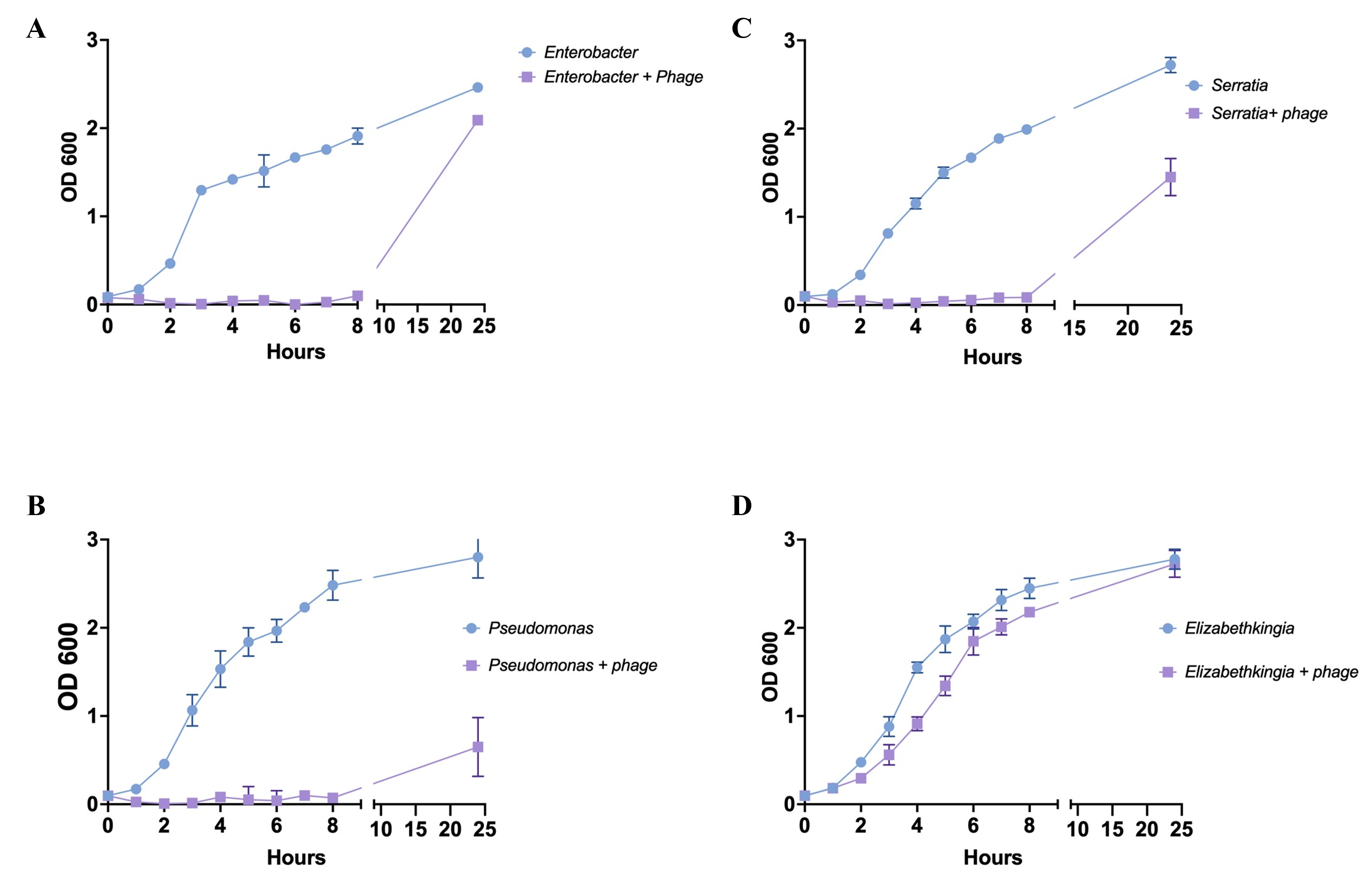

Supplement: FIG S2 [file mbio.03017-22-s0002.tif]

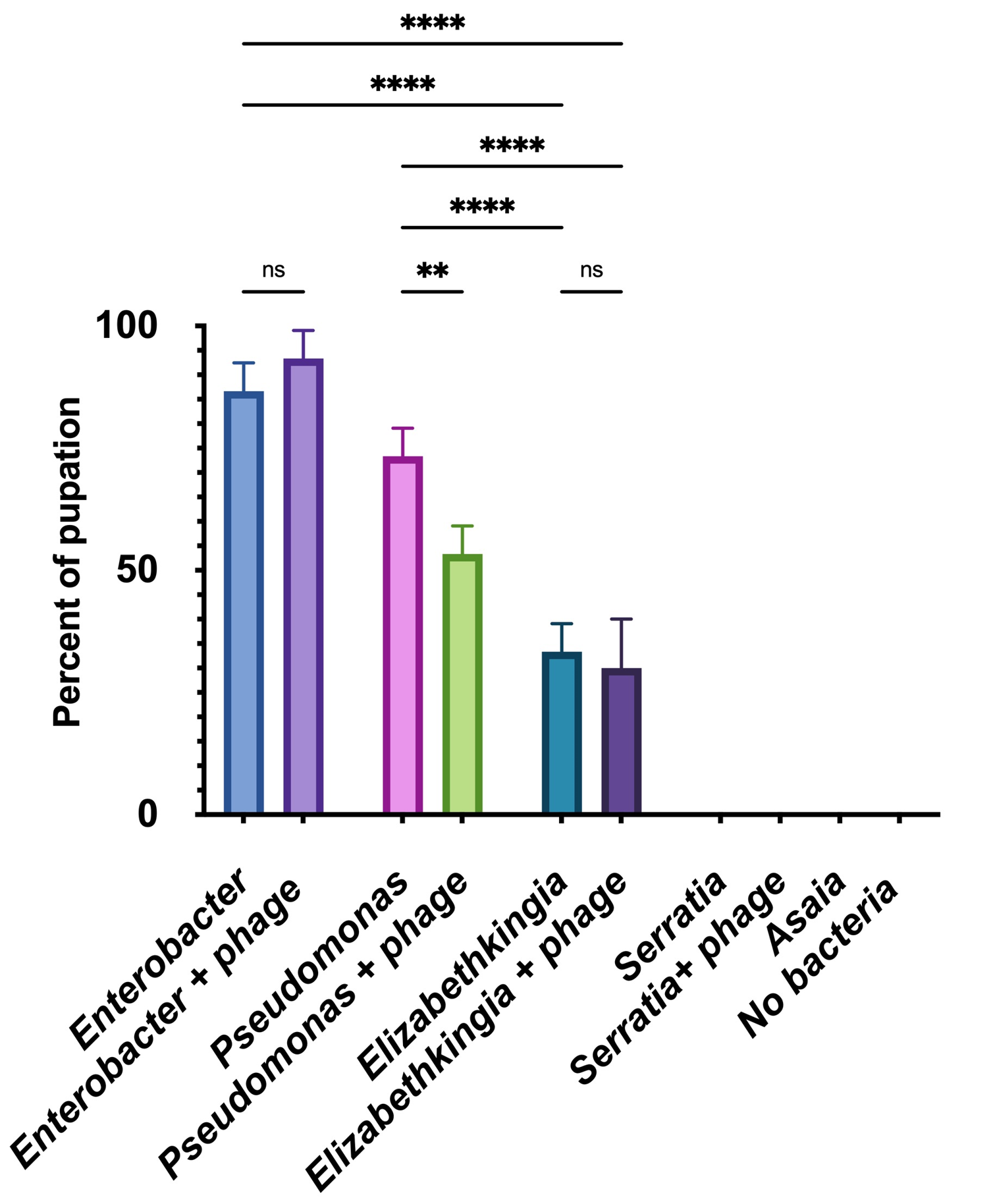

Supplement: FIG S3 [file mbio.03017-22-s0003.tif]

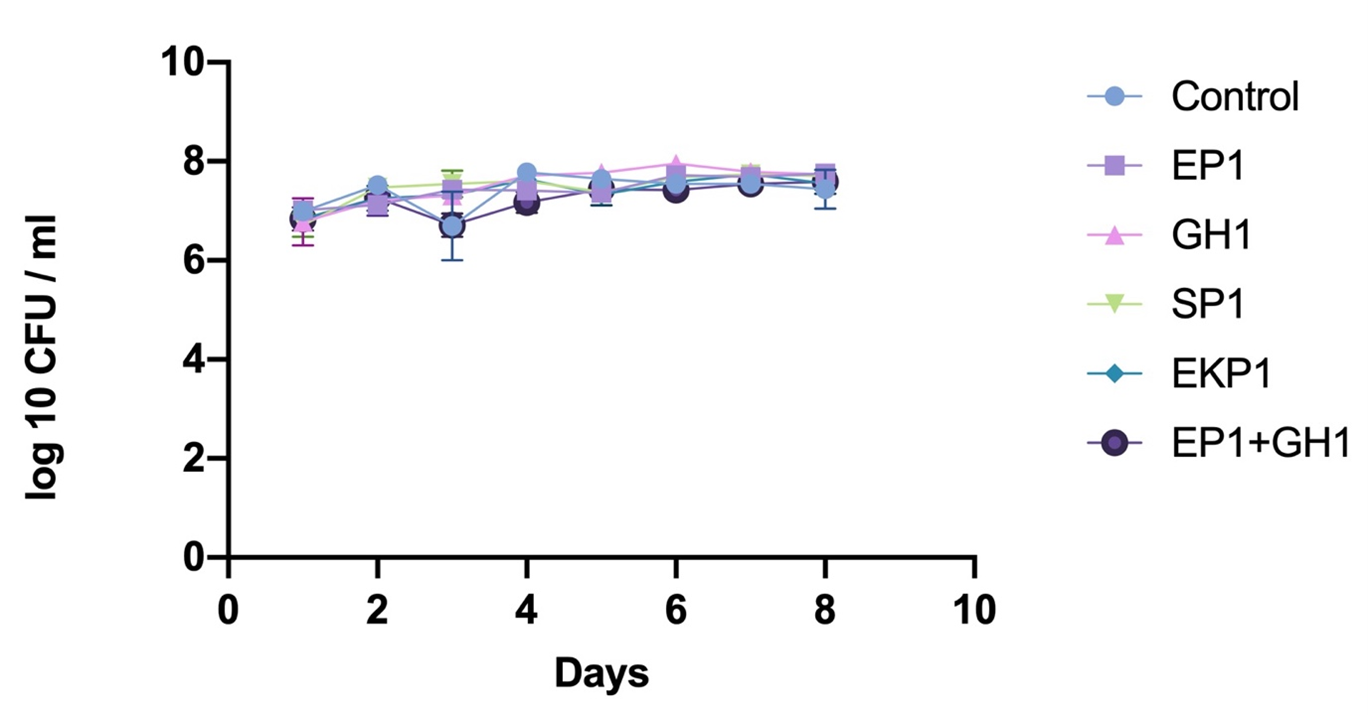

Supplement: FIG S4 [file mbio.03017-22-s0004.tif]
